# A Review of Copper Amalgam

**Published:** 1891-03

**Authors:** Julien W. Russell

**Affiliations:** Brooklyn, N. Y.


ARTICLE III.

### A REVIEW OF COPPER AMALGAM.

JULIEN W. RUSSELL, M. D. S., BROOKLYN, N. Y.

[Read before the Union Meeting in Boston, October 29, 1890.]

Probably never, in the history of the profession, has there been so much controversy excited in regard to the merits of a preparation, as in the case of copper amalgam.

It has been denounced by some as entirely unfit to be placed in the oral cavity; and upheld by others as the only filling material to use.

Tests have been made proving that it is affected by the oral secretions, and in time wastes away so as to render the filling useless. Equally reliable tests have proved that it is probably the most perfect filling material that has ever been invented.

These apparently contrary results have been obtained by men of equal ability and discernment, whose opinions have always been regarded as final. Later on we will try to prove that these conclusions are not quite as antagonistic as they appear.

It is now about three years since copper amalgam first became widely known in the United States, although it has been used abroad for over fifty years.

The sudden abandonment of alloys in favor of copper amalgam, and the enormous amount used for a while, is only another instance of the tendency of certain members of the profession to blindly adopt a new theory or remedy without thoroughly testing its merits. Consequently, while a method or material may be beneficial for certain cases under certain conditions, they immediately conclude that it is a panacea, and use it indiscriminately. The inevitable result is that there are necessarily many failures to record; and with that soundness of judgment and wideness of vision, for which the members of the dental profession are noted, these enthusiasts suddenly proclaim, that the theory or material is worthless, and condemn it as strongly as they formerly upheld it.

In order to arrive at definite conclusions, I entered into correspondence with dentists in different sections of the country, and taking the records of their experiments, I have compared them with some made by myself. The result I have the honor to present to you to-day.

Fault has been found with copper amalgam for many reasons; its opponents claim that it possesses no antiseptic properties; that in certain mouths it is soluble, and that not only do the detached particles discolor the adjoining teeth, but also there is great danger of constitutional derangements being produced from their absorption. In some persons very susceptible to the influences of mercury, salivation may result from its use, and in others copper poisoning.

It is said that the absorption of these minute particles of amalgam into the system would counteract the effects of drugs administered for diseases; and in throat and stomachic troubles would not only accelerate the disease, but so antagonize the relieving remedies that a cure would be impossible so long as the fillings remained in the mouth.

It is also claimed that copper amalgam will shrink, allowing moisture to penetrate between the filling and the walls of the cavity, resulting in a deep discoloration of the tooth.

Then, again, there is said to be a great wasting away at the cervical wall, *some* declaring that in a few months the filling becomes so thorougly softened at this point as to allow of its being removed with but little effort, and that when replaced the process is repeated in a very short time.

In favor of copper amalgam, it may be stated that a large number of tests, under all manner of conditions, have fully proven that it does not shrink to any appreciable extent.

In a steel mould one inch square and one-half an inch deep with absolutely vertical walls, a layer of copper amalgam one-eighth of an inch was carefully packed. After setting it was placed in aniline dye for a few days. When separated no traces of leakage were perceptible, and though the experiment was often repeated, the only signs of leakage found were when the amalgam had been used too dry, a thoroughly homogeneous mass not having been secured; and again, when the amalgam was so soft that it curled away from the walls.

The results of these experiments have been confirmed by some made in England, where the shrinkage was tested by a micrometer and found imperceptible when the amalgam was mixed to a medium consistency.

The discoloration of tooth structure may result from any one of the following causes:

The first is poor manipulation, which results in a leaky filling, as illustrated above.

The second is the teeth of poor structure are permeable by moisture, resulting in oxidation of the fillings, and this is not nearly so great as in the former case, and is much to be desired, as the oxide of copper hardens the tooth substance.

The third cause of discoloration is an impure material. A large number of the amalgams are made by precipitating the copper by means of zinc or iron; and, according to Dr. Mitchell of London, these form double sulphides which will blacken any tooth structure with which they come in contact. Take a hard dense tooth with a pure material, a careful insertion of the filling, and I doubt if any discoloration will be found.

The wasting away of carefully inserted fillings is certainly very discouraging, and especially so when a large crown filling is found hollowed out after a few months' use. The cause is generally to be found in an unusual acid condition of the saliva, and the only remedy is to cover the filling over with gold or an alloy.

In cases where the cervical part only is softened, it will be noticed that the teeth are afflicted by the peculiar erosion along the gum margins caused, as it is supposed, by acid mucus; in fact I have never heard of a case where this was not present. One thing is certain no wasting away has ever been found when the secretions were alkaline or neutral.

The theory that the dissolved particles affect the system, is very difficult to substantiate. The quantity that each day enters the system is so infinitesimal, and the question as to whether it is absorbed or passes through the system unchanged, is so unsettled, that no one can speak definitely on this point until we can obtain more accurate reports. I do not think that this objection need have any weight.

Everyone who has success with copper amalgam makes this statement: "It must be used with judgment, and more care is required than with alloys, not only in the manipulation of the material, but in inserting the filling.

To secure the best results from the use of copper amalgam, it should be remembered that its true position as a tooth saver is not in competition with gold, alloy or cement, but it has a place by itself, and that is where experience has shown the majority of other fillings have failed.

On the buccal surfaces and in large approximal cavities of the molars, especially where they extend under the margin of the gum. For cavities beyond the reach of the tooth-brush, and as a lining for very frail teeth, it is unsurpassed; and finally for preserving the deciduous and six-year molars in the mouths of children. This is the true position of copper amalgam.

Its manipulation is very simple: heat it slightly, grind vigorously; for the more it is ground the better; when amalgamated, squeeze out any surplus mercury until the mass is of the consistency of an ordinary alloy. In inserting the filling use small pieces, and burnish them from the centre to the sides of the cavity; when filled smooth the surface very gently and do not use the burnisher too much but draw it lightly from the center to the edge of the cavity; then allow it to harden, and finish in the usual manner.

In compound fillings of gold and copper amalgam, the method of adding the gold while the amalgam is still soft, is *not* to be commended; for the mercury that amalgamates with the gold does it at the expense of the copper; this part of the filling is apt to be porous, and in addition the close combination is likely to cause electric shocks. A better plan is to insert the amalgam and cover it with a temporary filling until it hardens; then cut the retaining points in the amalgam and complete with gold. Such a filling, when polished, presents a close line of union, and will preserve the tooth far better than when inserted as before described. If the moisture is thoroughly removed from the cavity that contains a certain amount of decayed tissue, and the amalgam placed over it, in time it will be found that decay is still progressing; but if a small quantity of moisture is left in the cavity, oxide of copper is found, and on account of its great antiseptic properties, further decay is prevented.

This is decidedly the best method for preserving the deciduous teeth; the only objection, darkening of the teeth, being of little consequence. Teeth filled in this manner three years ago, are still perfect, and from all appearances, will last as long as they are needed.

I have carefully tried to mark out the lines by which success with copper amalgam can be achieved. It does not offer any reward for poor workmanship, but on the contrary, requires the highest skill and judgment to obtain the best results. The future for it in this country is very great, and in time it will be recognized as one of our most valuable materials with which to preserve the teeth.—*Dental Mirror*.

